# The Development of AXL Inhibitors in Lung Cancer: Recent Progress and Challenges

**DOI:** 10.3389/fonc.2022.811247

**Published:** 2022-03-03

**Authors:** Yun Beom Sang, Joo-Hang Kim, Chang-Gon Kim, Min Hee Hong, Hye Ryun Kim, Byoung Chul Cho, Sun Min Lim

**Affiliations:** ^1^ Medical Oncology, Department of Internal Medicine, CHA Bundang Medical Center, CHA University School of Medicine, Seongnam, South Korea; ^2^ Division of Medical Oncology, Department of Internal Medicine, Yonsei Cancer Center, Yonsei University College of Medicine, Seoul, South Korea

**Keywords:** AXL, AXL inhibitor, resistance, targeted therapy, immunotherapy

## Abstract

AXL, along with MER and TYRO3, is a receptor tyrosine kinase from the TAM family. Although AXL itself is not thought to be a potent oncogenic driver, overexpression of AXL is known to trigger tumor cell growth, survival, invasion, metastasis, angiogenesis, epithelial to mesenchymal transition, and immune suppression. Overexpression of AXL is associated with therapy resistance and poor prognosis. Therefore, it is being studied as a marker of prognosis in cancer treatment or as a target in various cancer types. Recently, many preclinical and clinical studies on agents with various mechanisms targeting AXL have been actively conducted. They include small molecule inhibitors, monoclonal antibodies, and antibody-drug conjugates. This article reviewed the fundamental role of AXL in solid tumors, and the development in research of AXL inhibitors in recent years. Emphasis was placed on the function of AXL in acquired therapy resistance in patients with non-small cell lung cancer (NSCLC). Since clinical needs increase in NSCLC patients with acquired resistance after initial therapy, recent research efforts have focused on a combination treatment with AXL inhibitors and tyrosine kinase inhibitors or immunotherapy to overcome resistance. Lastly, we deal with challenges and limitations encountered in the development of AXL inhibitors.

## Introduction

AXL, first reported by Bryan at al. in 1991, is a protein coding gene separated from human myeloid leukemia cells (1). AXL, a receptor tyrosine kinase, consists of a transmembrane: an intracellular and an extracellular domain. Two fibronectin type-III and two immunoglobulin-like motifs make up the AXL extracellular domain (2). The kinase domain is phosphorylated by dimerization when growth arrest-specific 6 (Gas6), an AXL ligand, binds to the receptor. Then, a cross-phosphorylation method activates downstream proteins and signaling cascades are triggered (3). As a ligand that activates the TAM receptors, there is Protein S along with Gas6 (4). Since Gas6 has the highest affinity with AXL among the TAM family, AXL/Gas6 pathway is mainly being studied in cancer (5).

**Figure 1:**
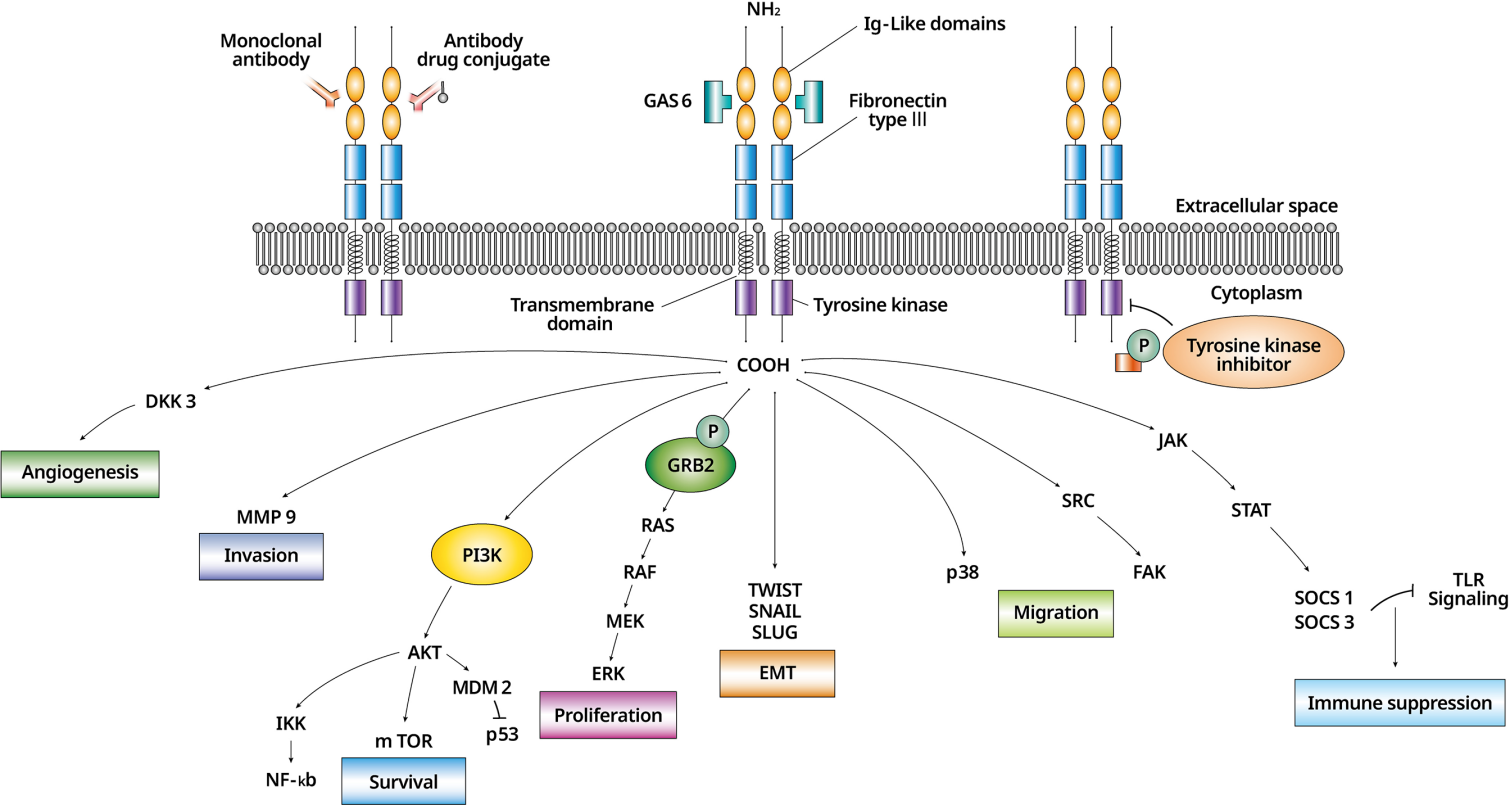
The biology of AXL/Gas6 signaling activation and downstream effectors in malignant cell. AXL/Gas6 activation and downstream signaling is shown. AXL promotes cell survival, proliferation, invasion, migration, angiogenesis, EMT, and immune suppression. An AXL inhibitor is expressed as a monoclonal antibody, an antibody-drug conjugate, and a tyrosine kinase inhibitor according to three different mechanisms. EMT, epithelial to mesenchymal transition; Gas6, growth arrest-specific 6.

Unlike other TAM receptors, AXL is involved in many cellular functions in variuos cell types (6). In cancer cells, AXL signaling changes tissue specifically through AXL dysregulation (7) (**Figure 1**). Activated AXL promotes phosphorylation of the growth factor receptor-bound 2 protein and mediates cell proliferation by activating the mitogen-activated protein kinase signaling pathway (8). AXL/Gas6 signaling is also involved in cell survival through the PI3K/AKT (phosphatidylinositol 3-kinase/protein kinase B) pathway (9) and activates pro-survival proteins including mouse double minute 2 homolog, inhibitor of NF-_K_B, and mammalian target of rapamycin (10). Steroid receptor coactivator (SRC) phosphorylated by AXL activates focal adhesion kinase (FAK) and induces the migration of cells (11). Migration is also conducted through p38 (3). Suppression of the SRC/FAK and p38 is related to decrease cell viability in the drug-resistant mesenchymal cells (11). Matrix metalloproteinase 9 is a required factor for AXL-mediated tumor invasion (12). Upregulated AXL increases the expression of the suppressor of cytokine signaling 1/3 through the Janus kinase/signal transducer and activator of transcription pathway, and down-regulation of Toll-like receptor signaling leads to immune suppression (13). AXL is involved in angiogenesis through Dickkopf-related protein 3 (14).

It has been shown that AXL is overexpressed in various carcinomas like NSCLC, breast cancer, gastric and colorectal cancer, and prostate cancer (15). Overexpression of AXL is known to be related to drug resistance by many PI3K-, ERK-, or epidermal growth factor receptor (EGFR)- inhibiting target agents (16). In NSCLC, higher AXL expression was seen in mesenchymal cancer cells than in epithelial cancer cells (17). AXL overexpression was dependent on the expression of vimentin, a marker for epithelial to mesenchymal transition (EMT), indicating that EMT may contribute to resistance acquisition (18, 19). Some studies showed that down-regulation of AXL can inhibit EMT progression and can induce a more increased response to TKI (20).

In order to overcome the occurrence of cancer and drug resistance, many studies on AXL inhibitors are being conducted. Many AXL inhibitors exist in the form of tyrosine kinase inhibitors; they are also being developed in the form of antibody-drug conjugate and monoclonal antibodies. AXL tyrosine kinase inhibitors can be categorized into two types depending on whether they bind to the active site of the DFG motif (type I) of AXL or not (type II) (21). As for AXL inhibitors, apart from single targets, dual or multi-targets like MET (mesenchymal-epithelial transition factor), VEGFR (vascular endothelial growth factor) are being developed (15). Monoclonal antibodies (mAbs) downregulate receptor expression or activation and downstream signaling by blocking the ligand of AXL (Gas6) (22). It has been reported that anti-AXL mAb promote the therapeutic effects of small molecule inhibitors against other targets such as EGFR, or VEGF, and also chemotherapy (23). An antibody-drug conjugate (ADC) is dependent on AXL expression but acts independently of activation of AXL/Gas6 signaling (24). Recently AXL-107- monomethyl auristatin E (MMAE) (24) and AXL-specific chimeric antigen receptor (CAR) (25) have also shown promising results in preclinical studies.

In lung cancer, AXL expression varies from ~33.0% to ~93.2% depending on known reports (26). In some reports, differences in AXL expression did not correlate with clinical outcomes (27). But many studies revealed that patients with NSCLC who exhibited high AXL mRNA expression showed poorer prognosis than patients exhibiting low AXL mRNA expression (28–30). And after using targeted therapy like EGFR inhibitor, increased AXL expression level was shown (31, 32). Considering that AXL’s genetic mutation and amplification are low in overall carcinoma (15), various studies are being conducted to find good biomarkers, such as phosphorylated AXL level (33).

Preclinical studies showed that the Gas6–AXL axis induced acquired resistance to the EGFR-tyrosine kinase inhibitors (TKI) erlotinib or osimertinib in NSCLC cells with EGFR-mutation positivity, and that AXL inhibitors combined with other drugs could overcome this resistance (34–36). Some clinical studies combining AXL inhibitors and immune checkpoint inhibitors (ICI) are ongoing in NSCLC patients who have failed therapy with ICI (37).

In this review, we introduced the progress of AXL inhibitors in recent years and updated the understanding of AXL inhibitors with a focus on lung cancer.

## Overview of Clinical Trial Landscape of AXL Inhibitors

We categorized the mechanisms of action into four groups: 1) antibody-drug conjugates (ADCs), 2) TKIs, and 3) mAbs, 4) Soluble receptors (Gas6 target). A list of selected AXL inhibitors according to the three categories are summarized in **Table 1**.

**Table 1:** A summary of clinical trials utilizing AXL inhibitors.

Drug	Target	Diseases	Phase	Recruiting status	NCT identifier
Antibody drug-conjugates					
Enapotamab Vedotin (HuMax-AXL-ADC)	AXL	Solid tumors (Including NSCLC)	I/II	Completed	NCT02988817
CAB-AXL-ADC alone or in combination with PD-1 inhibitor	AXL	Solid tumors (Including NSCLC)	I/II	Recruiting	NCT03425279
Tyrosine kinase inhibitors					
BGB324 + Docetaxel	AXL	NSCLC	I	Recruiting	NCT02922777
SLC-391	AXL	Solid tumors	I	Recruiting	NCT03990454
DS-1205c	AXL	NSCLC	I	Completed	NCT03255083
					NCT03599518
ASP2215 (Gilteritinib)	AXL, FLT3	Advanced solid tumors	I	Completed	NCT02456883
BPI-9016M	AXL, MET	Advanced solid tumors	Ia	Completed	NCT02478866
		C-MET dysregulated advanced NSCLC	Ib	Recruiting	NCT02929290
INCB081776	AXL, MER	Advanced solid tumors	Ia/Ib	Recruiting	NCT03522142
PF-07265807	AXL, MER	Advanced or metastatic solid tumors	I	Recruiting	NCT04458259
Q702	AXL, MER, CSF1R	Advanced solid tumors	I	Recruiting	NCT04648254
RXDX-106	AXL, TYRO3, MER, MET	Advanced solid tumors	I	Terminated	NCT03454243
TP-0903	AXL, MER, JAK2, ALK, ABL, Aurora A&B	Solid tumors (EGFR+ NSCLC)	I	Active, not recruiting	NCT02729298
MGCD265	AXL, MET, RON, VEGFR1/2/3,	NSCLC with activating genetic alterations in MET	II	Completed	NCT02544633
MGCD516	AXL, MET, EPHA3, RET, VEGFR3, PDGFR, KIT, TRK family, DDR2	Advanced solid tumors	I/Ib	Active, not recruiting	NCT02219711
Monoclonal antibodies					
YW327.6S2	AXL	NSCLC, breast cancer	Preclinical	YW327.6S2 enhances the anti-tumor effect of erlotinib in an A549 xenograft model	
D9 & E8	AXL	Pancreatic cancer	Preclinical	Anti-neoplastic effect of D9 and E8 in both cellular and animal xenograft pancreatic tumor models	
MAb173	AXL	Kaposi sarcoma, Renal cell carcinoma	Preclinical	*In vivo* xenograft studies, MAb173 reduced tumor growth, increased tumor cell apoptosis, and markedly decreased AXL protein levels in tumors. hMAb173 significantly induced RCC cell apoptosis in histoculture and inhibited the growth of RCC tumor *in vivo* by 78%.	
Soluble receptors (Gas6 target)					
AVB-S6-500	Gas6	Ovarian cancer	I/II	Active, not recruiting	NCT03639246
		Ovarian cancer, Fallopian tube cancer, Peritoneal cancer	I/II	Active, not recruiting	NCT04019288
		Ovarian cancer	III	Recruiting	NCT04729608
		Renal cell carcinoma	I/II	Recruiting	NCT04300140
		Advanced pancreatic adenocarcinoma	I/II	Recruiting	NCT04983407

NSCLC, non-small cell carcinoma; EGFR, epidermal growth factor receptor; RCC, renal cell carcinoma.

### Antibody-Drug Conjugates (ADCs)

#### Enapotamab Vedotin (HuMax-AXL-ADC)

Enapotamab vedotin is an AXL-targeted ADC created by combining human AXL-targeted immunoglobulin G1 with MMAE, the microtubule-destroying agent. In the preclinical study, enapotamab vedotin (2 and 4mg/kg) exhibited anti-tumor effects in osimertinib-resistant patient-derived xenograft (PDX) models (38). The study evaluating the safety of enapotamab vedotin (HuMax-AXL-ADC) treatment is an ongoing phase I/II study of solid tumors, including NSCLC (NCT02988817).

#### Conditionally Active Biologic Anti-AXL Antibody Drug Conjugate (CAB-AXL-ADC)

CAB-AXL-ADC binds selectively to human and cyno AXL expressing cells in the tumor microenvironment but has reduced binding under normal tissue conditions. CAB-AXL-ADC demonstrated the ability to induce cytotoxicity of human tumor cell lines expressing AXL *in vitro*, and inhibit tumor growth in lung, prostate, pancreatic human tumor xenografts, and in selected gemcitabine-resistant pancreatic cancer-derived xenograft models, *in vivo* (39). A phase I trial using CAB-AXL-ADC alone in advanced solid tumor patients is ongoing, as well as a phase II trial combining CAB-AXL-ADC with PD-1 (programmed cell death protein 1) inhibitors (NCT03425279). In addition, a separate phase II study is being conducted only for NSCLC participants (NCT04681131). These studies are yet to report interim results.

### Tyrosine Kinase Inhibitor (TKI)

#### BGB324 (Bemcentinib, R428)

BGB324 is a small molecule AXL TKI. BGB324 revealed AXL inhibitory activity (EC_50_/IC_50_, 14 nmol/L) in both an *in vitro* biochemical kinase assay and a cell-based assay in HeLa cells reflecting AXL signaling (40). BGB324 inhibited the appearance of EMT-related EGFR inhibitor resistance in NSCLC xenografts in a preclinical study (41).

In another study, BGB324 inhibited the aggressiveness of PDA cells (pancreatic cancer) *in vitro* and enhanced gemcitabine efficacy *in vivo* (42). Phase I and II clinical studies of BGB324 are ongoing in non-small cell carcinoma, in which combinations with other agents such as docetaxel (NCT02922777), erlotinib (NCT02424617), and pembrolizumab (NCT03184571) are also being investigated.

#### SLC-391

A small molecule inhibitor, SLC-391 has potent efficacy against many cancer cell lines, by inhibiting AXL/PI3K/AKT-dependent cell proliferation and survival (50 mg/kg p.o, CT-26 murine colon carcinoma cell line). In addition, a synergistic anti-tumor effect was observed when a high PD-1 expression CT-26 syngeneic model was treated with a combination of SLC-391 and a PD-1 inhibitor, and the overall survival rate of the combination group was dramatically prolonged, in comparison with the vehicle control group (43). A phase I study of SLC-391 in solid tumors is ongoing (NCT03990454).

#### DS-1205c

A novel AXL inhibitor, DS-1205c is another sulfate hydrate, with similar stoichiometries to DS-1205b. In a preclinical study, DS-1205b intensely suppressed hGas6-dependent cell migration *in vitro* and showed potent anti-tumor effects in AXL-overexpressing NSCLC xenograft cells *in vivo* (IC50, 1.3 nM) (44). A phase I clinical study of DS-1205c plus EGFR TKI (gefitinib) is ongoing (NCT03599518). The study of DS-1205c plus EGFR TKI (osimertinib) was terminated based on a business decision by the sponsor (NCT03255083).

#### ASP2215 (Gilteritinib)

ASP2215 is a highly selective, dual AXL/FLT3 (FMS-like tyrosine kinase 3) inhibitor that showed anti-leukemic activity in relapsed or refractory acute myeloid leukemia (AML) patients. In the preclinical study, ASP2215 regressed the tumor size and decreased proliferation in FLT3 mutated cells and AML xenograft models (IC50, 0.29 nM and 0.73 nM, respectively (45). A study combining ASP2215 and erlotinib in EGFR-positive NSCLC patients after EGFR inhibitor use was terminated due to adverse events related to the combination therapy (NCT02495233). Reported SAEs (serious adverse events) were alanine aminotransferase increase (n=4), aspartate aminotransferase increase (n=3), renal failure-acute (n=1), and pleural effusion (n=1) in 10 subjects.

#### BPI-9016M

A dual AXL/c-Met inhibitor, BPI-9016M potently regressed tumor size in NSCLC PDX models. The effect was greater in high c-Met expression tumors (IC50 ranging from 5.3 μM to 27.1 μM) (46). BPI-9016M showed favorable safety and pharmacokinetic profiles in a phase I clinical trial involving 20 Chinese NSCLC patients. Grade 3 or higher treatment-related adverse events (TRAEs) reported during treatment included hypertension (15%), laryngitis (5%), and pulmonary thromboembolism (PTE) (5%). Dose-limiting toxicity (DLT) was not detected, and the maximum tolerated dose was not found. Among 19 patients, one showed a partial response (PR) and 10 showed stable disease (SD) (NCT02478866). A phase I clinical trial of BPI-9016M in c-Met dysregulated NSCLC patients is also underway (NCT02929290).

#### INCB081776

INCB081776 is a novel AXL/MER inhibitor that increases anti-tumor immune activity. In preclinical data, INCB081776 potently inhibited the recombinant AXL/MER enzymes (IC50, 0.61 nM and 3.17 nM against AXL/MER, respectively) (47). A phase I study of INCB081776 in advanced solid tumors is ongoing (melanoma, NSCLC, squamous head & neck cancer, soft tissue sarcoma) (NCT03522142).

#### PF-07265807

PF-07265807 is an AXL/MER inhibitor that enhances the function of dendritic cells to cross-prime CD8+ T cells. In preclinical assays, PF-07265807 alone revealed anti-tumor effects and showed increased cure rates in tumor models when combined with PD-1 inhibitor (48). A phase I clinical trial of PF-07265807 in advanced solid malignancies is ongoing (NCT 04458259).

#### Q702

Q702 is an AXL, MER and CSF1R kinase inhibitor. A study group presented the potential of Q702 leading to tumor regression through immune stimulating activity by decreasing T-reg cells, M2 macrophages, and myeloid-derived suppressor cells and promoting antigen presentation and direct cytotoxic activity in syn-tumor models including AML and EGFR TKI resistant NSCLC (49). The same group presented the anti-tumor efficacy and immune mechanism of Q702, in combination with anti-PD-1, in the various syngeneic models in a recent report (50). A phase I clinical trial of Q702 in advanced solid malignancies is underway (NCT04648254).

#### RXDX-106 (CEP-40783)

RXDX-106 is a pan TAM RTK (receptor tyrosine kinase) family inhibitor (IC50 of TYRO3, 3.50 nM; AXL, 0.69 nM, MER: 1.89 nM). In wild-type mice, RXDX-106 treatment leads to tumor growth inhibition, but not in immuno-deficient mice. RXDX-106 also increases the effects of ICIs, resulting in enhanced anti-tumor efficacy and survival (51). A clinical trial of RXDX-106 in advanced solid tumors for which standard therapy was not effective was terminated by the sponsor (NCT03454243).

#### TP-0903

TP-0903 is an AXL inhibitor (IC50, 27 nM *in vitro*) (52, 53). TP-0903 inhibits AXL phosphorylation and modifies EMT. TP-0903 reduces anti-apoptotic proteins like BCL-2, MCL-1, and XIAP, and enhances dose-dependent chronic lymphocytic leukemia (CLL) cell death (54, 55). A phase I study of TP-0903 in advanced solid malignancies, including EGFR-mutation NSCLC patients is ongoing (NCT02729298).

#### MGCD265 (Glesatinib)

MGCD265 is a multi-target inhibitor, suppressing tumor cell growth, survival, and angiogenesis (56). In an *in vitro* study, MGCD265 showed potent inhibition of c-Met/VEGFR/Tie-2/Ron action, with IC50s in the nano-molar range (57). A phase II study of MGCD265 in Met-altered NSCLC patients was conducted, although efficacy data were not statistically significant in the results. Dehydration (6/68), pneumonia (5/68), and myocardial infarction (2/68) were shown in the SAE report (NCT02544633).

#### MGCD516 (Sitravatinib)

MGCD516 is a small molecule inhibitor targeting multiple RTKs. In a preclinical study, MGCD516 showed potent anti-tumor effects and had better efficacy compared to imatinib and crizotinib in different sarcoma models (56). A phase III trial combining MGCD516 and nivolumab in advanced NSCLC patients is currently underway (NCT03906071).

### Monoclonal Antibodies (mAbs)

#### YW327.6S2

YW327.6S2 is a mAb for AXL, which inhibits receptor activation and downstream signaling. In a preclinical study, YW327.6S2 decreased tumor size and increased the anti-VEGF treatment efficacy in NSCLC and breast cancer xenograft models. YW327.6S2 elevated the anti-tumor activity of erlotinib and chemotherapy in NSCLC models. The study group showed that AXL mAbs affect tumor cells and stroma by modulating tumor-associated vasculature and immune cell functions (23). Research on this drug is in the preclinical stage.

#### D9 and E8

D9 and E8 are selective anti-AXL mAbs. They inhibit Gas6-induced phosphorylation of AXL and downstream signaling. D9 and E8 induced the suppression of Gas6-mediated phosphorylation of AXL and conducted *via* receptor down-regulation and internalization irrespective of Gas6 in pancreatic cancer cells and xenograft models (58). Research on this drug is in the preclinical stage.

#### MAb173

MAb173 inhibited Kaposi sarcoma cell invasion by inducing receptor degradation *in vitro* (59). In a preclinical study, irrespective of Kaposi sarcoma associated herpes virus infection, MAb173 reduced tumor size and down-regulated AXL protein levels in Kaposi sarcoma tumor cells. These results showed that AXL has an important role in Kaposi sarcoma pathogenesis (59). In another *in vivo* study, MAb173 potently increased Renal cell carcinoma (RCC) cell apoptosis and decreased RCC tumor size by 78% (60). Research on this drug is in the preclinical stage.

### Soluble Receptors (Gas6 Target)

#### AVB-S6-500

An AXL decoy receptor, AVB-S6-500 is a soluble receptor to bind Gas6 ligand. In a preclinical study of ovarian cancer and renal cell carcinoma, AVB-S6-500 reduced Gas6-induced AXL phosphorylation and tumor growth (61, 62). Many clinical studies of AVB-S6-500 in ovarian cancer (NCT03639246, NCT04019288, NCT04729608), renal cell carcinoma (NCT04300140) and pancreatic cancer (NCT04983407) are ongoing.

## AXL Inhibitors in Lung Cancer

In this section, we will review ongoing clinical trials using AXL inhibitors in lung cancer as shown in **Table 2**. AXL inhibitors are being investigated in two settings. The first is the setting of acquired resistance after EGFR inhibitor use. The EGFR T790M mutation is the most frequently cause of resistance after initial 1^st^ or 2^nd^ generation EGFR TKI use in metastatic EGFR-mutant NSCLC patients. Up-regulation of the bypass signaling pathway is also an important resistance mechanism. Up-regulation of AXL expression levels has been also shown in EGFR-mutant NSCLC patients who developed resistance to erlotinib treatment. Some xenograft studies of AXL inhibitors combined with EGFR TKI showed meaningful results in overcoming such resistance (44, 63). AXL overexpression was observed with an EMT-like feature in transcriptomic analyses of NSCLC cell lines (17). This also implies that targeting AXL could overcome acquired resistance after EGFR TKI use related to EMT. Second, AXL inhibitors are being explored in combination with ICIs due to their immune-modulatory effects. Although ICIs have emerged as promising anti-tumor agents, there still exists a need to overcome the resistance developed after using them and to find treatment combinations with synergistic mechanisms of action. AXL is also related to resistance to cytotoxic T-cell mediated cell death (64). In a previous preclinical study, an AXL inhibitor made the cells more sensitive to T-cell-mediated killing (65). In various types of solid tumors, overexpression of AXL has been shown after PD-1 inhibitor failure (15). In preclinical models of NSCLC, AXL inhibition has shown a synergistic effect in combinations with ICIs (37).

**Table 2:** Clinical studies combining AXL inhibitor with other drugs in lung cancer.

Drug	Diseases	Phase	Status	Results	NCT identifier
**TKI combination**					
BGB324 (Bemcentinib) + Erlotinib	Stage IIIb or IV NSCLC (has been receiving erlotinib)	I/II	n=32	In the run-in arm, 2/8 pts achieved SD for 1 yr, (19% tumor shrinkage in 1 pt). In arm A, 1/8 pts achieved tumor shrinkage of 38%. A further 5 pts reported SD. In arm B (11 pts), one achieved a PR and one a SD on the combination; mPFS was 1.4 mths. In arm C, 11/13 pts were evaluated for efficacy. 1 PR was reported, with 47% tumor shrinkage; 9 other pts achieved SD. mPFS is 12.2 mths. Treatment was generally well-tolerated.	NCT02424617
DS-1205c + Gefitinib	Metastatic or unresectable EGFR-mutant NSCLC (ADC) (has been receiving erlotinib, gefitinib, afatinib, or osimertinib)	I	n=21	Dose Escalation in Cohort 1 (200 mg BID; n=5), Cohort 2 (400 mg BID; n=4), Cohort 3 (800 mg BID; n=6), Cohort 4 (1,000 mg BID; n=1), and Cohort 5 (1,200 mg BID; n=4); 1 pt in Cohort 3 and 1 pt in Cohort 5 experienced DLT, and RDE was determined as 800 mg BID. There have been no SAEs directly related to DS-1205c.	NCT03599518
DS-1205c + Osimertinib	Metastatic or unresectable EGFR-mutant NSCLC (ADC) (has been receiving erlotinib, gefitinib, afatinib; or is currently receiving osimertinib)	I	n=13	This study was terminated based on a business decision by the sponsor. According to their interim report, 9 SD, 3 PD, 1 NE were reported. Frequent TEAEs included elevated liver enzyme, vomiting, and fatigue.	NCT03255083
ASP2215 (Gilteritinib) + Erlotinib	Advanced NSCLC who have acquired resistance to an EGFR TKI	Ib/II	n=10, terminated	All subjects in both arms Gilteritinib 120 mg + Erlotinib 150 mg (n=3), Gilteritinib 80 mg + Erlotinib 150 mg (n=7) showed drug-related TEAEs. SAEs were increased ALT (n=4), increased AST (n=3), renal failure acute (n=1), and pleural effusion (n=1).	NCT02495233
ONO-7475 + Osimertinib	AXL-overexpressing EGFR-mutated NSCLC xenograft models	preclinical			
**ICI combination**					
BGB324 + Pembrolizu mab	Advanced NSCLC (ADC) (has disease progression after platinum containing CTx or anti-PD-(L)1- therapy	II	n=24	14 were ongoing; 6 of 10 pts who had reached their first scan. 3 pts with PR. 2 pts had SD. There were no G4 TRAEs.	NCT03184571
MGCD265 (Glesatinib) + Nivolumab	Advanced or metastatic NCSLC (prior treatment with a ICI)	II	Active, not recruiting	Not available	NCT02954991
MGCD516 (Sitravatinib) + Nivolumab	Advanced or metastatic NCSLC (prior treatment with a ICI)	II	Active, not recruiting	Enrolled 11 pts and 6/11 pts have had at least one on-study tumor assessment. 2 pts out of 6 have achieved PR. Treatment has been associated with manageable side effects to date.	NCT02954991
MGCD516 (Sitravatinib) + Tislelizumab	Locally advanced or metastatic NCSLC	III	Not recruiting	Not available	NCT04921358
MGCD516 (Sitravatinib) + Pembrolizu mab	Advanced NSCLC (prior ICI is not allowed)	II	Not recruiting	Not available	NCT04925986
CAB-AXL-ADC (BA3011) + Nivolumab	Metastatic NSCLC (had disease progression on PD-(L)1-therapy)	II	Recruiting	Not available	NCT04681131

NSCLC, non-small cell carcinoma; pts, patients; SD, stable disease; pt, patient; PR, partial response; mPFS, median progression free survival; ADC, adenocarcinoma; mths, months; BID, twice a day; PD, progressive disease; NE, not evaluable; DLT, dose-limiting toxicity; RDE, recommended dose for expansion; SAEs, serious adverse events; TEAEs, treatment emergent adverse events; ICI, immune checkpoint inhibitor.

### TKI Combination

#### BGB324 (Bemcentinib) + Erlotinib

The results of a BGB324 clinical trial in advanced NSCLC patients were recently reported (NCT02424617). In phase I, BGB324 alone and BGB324 plus erlotinib (in patients who previously progressing on erlotinib, arm A) were evaluated. In phase II, patients who previously had progression after any EGFR inhibitor use (arm B) or who were responding/stable on erlotinib treatment in the first line setting (arm C) were treated with BGB324 plus erlotinib. In the run-in arm, two-eights of the patients achieved SD for 1 year, including 19% tumor shrinkage in one patient. In arm A, one-eighth of the patients achieved a tumor shrinkage of 38%, with treatment duration of 2 years until progression. Five patients additionally reported SD. In arm B, one achieved a PR and one a SD on the combination; durations of treatment were 1 year, and 6 months, respectively. None of these 2 patients had EGFR T790M mutation. Median progression free survival (mPFS) was 1.4 months. In arm C, 11/13 patients were evaluable for efficacy. One PR was reported with 47% tumor shrinkage; duration of treatment was 315 days. Nine other patients achieved SD; mPFS is currently 12.2 months. Treatment was tolerable. Common TRAEs (> 20% of patients) were diarrhea (70%; Grade 3, 20%), nausea (50%; Grade 3, 0%), QTc prolongation (35%; Grade 3, 3%), vomiting (35%; Grade 3, 0%), and fatigue (25%; Grade 3 5%). One unrelated Grade 4 and no treatment-related deaths were reported. Bemcentinib with erlotinib combination is feasible and tolerable in NSCLC patients. A benefit was seen in a subset of patients who showed progression after EGFR TKI use or were taking erlotinib concurrently in remission, in the first line (66).

#### DS-1205c + Gefitinib

A phase I study of the AXL inhibitor DS-1205c plus gefitinib in advanced EGFR-mutant NSCLC patients is currently being conducted (NCT03599518). Patients who showed progression on EGFR TKI and did not have mutation were eligible. A dose-escalation cohort (200-1200 mg twice a day, BID) was completed in April 2020. One patient receiving 800 mg BID had DLT, so the recommended dose for expansion was 800 mg BID. SAEs caused by DS-1205c were not observed. One patient continued with SD for more than three months (67).

#### DS-1205c + Osimertinib

A phase I study of the AXL inhibitor DS-1205c plus osimertinib in advanced EGFR-mutant NSCLC patients was initiated April 10, 2019, but has been terminated based on a business decision by the sponsor (NCT03255083). According to their interim report, of a total 13 enrolled patients, nine patients were SD, three patients were PD and one patient was not evaluable. Frequent TEAEs included elevated liver enzyme, vomiting, and fatigue.

#### ASP2215 (Gilteritinib) + Erlotinib

According to the final report of Astellas (ISN 2215-CL-5101, no publications based on the results of this study), 10 patients were enrolled in the study of phase Ib/II combining ASP2215 and erlotinib in EGFR-positive advanced NSCLC patients who showed progression after EGFR inhibitor use. Since only two patients received more than two cycles of study treatment, there was not enough data to allow for meaningful results regarding the efficacy of this combination regimen. This study demonstrated that ASP2215 in combination with erlotinib at the dose levels evaluated did not show an acceptable safety profile due to increased hepatic enzymes.

#### ONO-7475 + Osimertinib

In a preclinical study, ONO-7475 (AXL/MER inhibitor) plus osimertinib overtly reduced tumor size and suppressed tumor growth compared to osimertinib alone, or combination therapy after acquisition of osimertinib resistance in AXL-overexpressing EGFR-mutated NSCLC xenograft models (68). In their study, cell line-derived xenograft (CDX) mice models and cell line–based analysis showed that the combination of ONO-7475 and osimertinib was more effective at the initial phase than at the osimertinib-acquired resistance phase in high-AXL–expressing EGFR-mutated NSCLC cells (68). Clinical trials are not yet in progress.

### ICI Combination

#### BGB324 (Bemcentinib) + Pembrolizumab

In a preclinical study, BGB324 was shown to increase the efficacy of a PD-1 inhibitor (69). A phase II clinical trial of BGB324 plus pembrolizumab for previously treated and immuno-therapy naive patients with stage IV lung adenocarcinoma is ongoing. According to their interim study report (38 patients; 24 and 14 in stage 1 and 2, respectively), the most frequent TRAEs were increased hepatic enzymes (37%), diarrhea (29%), and asthenia (17%). With the use of corticosteroids, TRAEs were well-managed. According to reports, among 29 evaluable patients, 7 patients (24%) showed PR. In AXL-positive patients, the objective response rate was 40%, mPFS was 5.9 months (mPFS of total patients was 4.0 months), and the median overall survival was not reached (70).

#### MGCD265 (Glesatinib) and MGCD516 (Sitravatinib) + Nivolumab

Glesatinib and sitravatinib revealed anti-tumor effects and increased PD-1 inhibition by enhancing an anti-tumor microenvironment (71). A phase II clinical trial in refractory NSCLC patients found that sitravatinib can induce recovery of response to nivolumab. Among six evaluable patients, two patients showed PR, and the treatment was well-tolerated (72).

## Discussion

Overexpression of the AXL gene is involved in resistance to anti-cancer therapeutics in various types of cancer. Therefore, development of AXL inhibitors is ongoing to overcome the acquired resistance that emerges after a favorable response to therapy. In lung cancer, especially NSCLC, EMT and bypass signal activation are known mechanisms of resistance after EGFR TKI use. In order to overcome this resistance, many clinical studies using AXL inhibitors are being conducted. AXL inhibitors are highly anticipated for lung cancer treatment in terms of their ability to inhibit EMT and regulate the tumor microenvironment. However, there are still many hurdles to overcome in order to incorporate AXL inhibitors into clinical practice. Since the effect of AXL inhibitor alone is not yet satisfactory, the bio-chemical understanding of the role of AXL in the treatment of lung cancer needs to be further progressed. Combining AXL inhibitor with EGFR TKI or immunotherapy is also a part of efforts to develop more effective treatment strategy. In terms of toxicity, some studies have reported SAEs such as increased hepatotoxicity, but finding an appropriate combination will be a challenge. Moreover, the expression level of the AXL gene is not yet optimized for use as a predictive biomarker for AXL inhibitors, and it is also important to set the cut-off of the AXL expression level properly for each cancer type. Ongoing clinical trials and future studies are warranted to develop more effective and safe treatment strategies utilizing AXL inhibitors, whether in combination or as a single-agent.

## Author Contributions

All authors contributed to manuscript revision, read, and approved the submitted version.

## Conflict of Interest

The authors declare that the research was conducted in the absence of any commercial or financial relationships that could be construed as a potential conflict of interest.

## Publisher’s Note

All claims expressed in this article are solely those of the authors and do not necessarily represent those of their affiliated organizations, or those of the publisher, the editors and the reviewers. Any product that may be evaluated in this article, or claim that may be made by its manufacturer, is not guaranteed or endorsed by the publisher.
